# Severe Silicosis in Engineered Stone Fabrication Workers — California, Colorado, Texas, and Washington, 2017–2019

**DOI:** 10.15585/mmwr.mm6838a1

**Published:** 2019-09-27

**Authors:** Cecile Rose, Amy Heinzerling, Ketki Patel, Coralynn Sack, Jenna Wolff, Lauren Zell-Baran, David Weissman, Emily Hall, Robbie Sooriash, Ronda B. McCarthy, Heidi Bojes, Brian Korotzer, Jennifer Flattery, Justine Lew Weinberg, Joshua Potocko, Kirk D. Jones, Carolyn K. Reeb-Whitaker, Nicholas K. Reul, Claire R. LaSee, Barbara L. Materna, Ganesh Raghu, Robert Harrison

**Affiliations:** ^1^National Jewish Health, Denver, Colorado; ^2^University of Colorado School of Medicine, Denver; ^3^Occupational Health Branch, California Department of Public Health; ^4^Epidemic Intelligence Service, CDC; ^5^Texas Department of State Health Services; ^6^Department of Medicine, University of Washington, Seattle; ^7^Department of Environmental and Occupational Health Sciences, University of Washington, Seattle; ^8^Colorado School of Public Health, Denver; ^9^Respiratory Health Division, National Institute for Occupational Safety and Health, CDC, Morgantown, West Virginia; ^10^Medical Surveillance Services, Concentra, Waco, Texas; ^11^Department of Pulmonary and Critical Care Medicine, Kaiser Permanente, Downey, California; ^12^Public Health Institute, Oakland, California; ^13^Division of Occupational and Environmental Medicine, Department of Medicine, University of California, San Francisco; ^14^Department of Pathology and Laboratory Medicine, University of California, San Francisco; ^15^Safety & Health Assessment & Research for Prevention, Washington State Department of Labor & Industries, Olympia; ^16^Office of the Medical Director, Washington State Department of Labor & Industries, Olympia.

Silicosis is an incurable occupational lung disease caused by inhaling particles of respirable crystalline silica. These particles trigger inflammation and fibrosis in the lungs, leading to progressive, irreversible, and potentially disabling disease. Silica exposure is also associated with increased risk for lung infection (notably, tuberculosis), lung cancer, emphysema, autoimmune diseases, and kidney disease (*1*). Because quartz, a type of crystalline silica, is commonly found in stone, workers who cut, polish, or grind stone materials can be exposed to silica dust. Recently, silicosis outbreaks have been reported in several countries among workers who cut and finish stone slabs for countertops, a process known as stone fabrication (*2*–*5*). Most worked with engineered stone, a manufactured, quartz-based composite material that can contain >90% crystalline silica (*6*). This report describes 18 cases of silicosis, including the first two fatalities reported in the United States, among workers in the stone fabrication industry in California, Colorado, Texas, and Washington. Several patients had severe progressive disease, and some had associated autoimmune diseases and latent tuberculosis infection. Cases were identified through independent investigations in each state and confirmed based on computed tomography (CT) scan of the chest or lung biopsy findings. Silica dust exposure reduction and effective regulatory enforcement, along with enhanced workplace medical and public health surveillance, are urgently needed to address the emerging public health threat of silicosis in the stone fabrication industry.

## Investigation and Results

**California**. In January 2019, the California Department of Public Health identified, through review of hospital discharge data for silicosis diagnoses (*International Classification of Diseases, Tenth Revision* [ICD-10] code J62.8), a Hispanic man aged 37 years who was hospitalized in 2017 (CA-1) (Table). He worked at a stone countertop fabrication company during 2004–2013, mainly with engineered stone. His work tasks included polishing slabs and dry-cutting and grinding stone edges. Workplace measurements during a California Division of Occupational Safety and Health inspection in 2009 showed respirable crystalline silica levels up to 22 times higher than the permissible exposure limit (PEL) of 0.1 mg/m^3^ in effect in California at that time.^†^ After developing respiratory symptoms in 2012, he had a chest CT scan, which revealed findings of silicosis. Pulmonary function testing showed restrictive defects with reduced diffusion capacity; surgical lung biopsy showed mixed dust pneumoconiosis with polarizable particles consistent with silica. He concurrently received a diagnosis of scleroderma, with positive anti-Scl-70 and antinuclear antibodies. He died from silicosis in 2018 at age 38 years.

**TABLE:** Demographic, occupational, and clinical features of 18 silicosis cases in stone fabrication workers — California, Colorado, Texas, and Washington, 2017–2019

State-Patient no.	Age range (yrs) at diagnosis	Decade of first exposure* (total yrs)	Chest CT abnormalities	Pulmonary function test findings (FEV_1_, FVC, and DLCO percentage predicted; FEV_1_/FVC ratio)	Other associated conditions
CA-1^†^	30–39	2000s (9 yrs)	Diffuse ground glass and solid centrilobular nodules; mediastinal lymphadenopathy	FEV_1_: 35%^§^	Scleroderma
				FVC: 33%^§^	
				FEV1/FVC: 86%	
				DLCO: 13%^§^	
CA-2^†,¶^	30–39	2000s (13 yrs)	Bilateral ground glass opacities and nodules	Not performed	Rheumatoid arthritis
CA-3	30–39	2000s (11 yrs)	Diffuse, upper lung predominant perilymphatic nodules	FEV_1_: 77%^§^	None
				FVC: 83%	
				FEV_1_/FVC: 76%	
				DLCO: 70%^§^	
CA-4	40–49	2000s (14 yrs)	Subpleural nodules with upper lobe predominance; mild mediastinal lymphadenopathy	FEV_1_: 73%^§^	None
				FVC: 79%^§^	
				FEV_1_/FVC: 75%	
				DLCO: 57%^§^	
CA-5	30–39	2000s (14 yrs)	Upper lobe architectural distortion and ground glass micronodules; mediastinal lymphadenopathy.	FEV_1_: 58%^§^	None
				FVC: 71%^§^	
				FEV_1_/FVC: 67%^§^	
				DLCO: 73%^§^	
CA-6	50–59	2000s (16 yrs)	Bilateral upper lobe fibronodular scarring; calcified mediastinal lymphadenopathy.	FEV_1_: 94%	None
				FVC: 96%	
				FEV_1_/FVC: 98%	
CO-1	40–49	2000s (12 yrs)	Upper lung predominant perilymphatic nodules	FEV_1_: 86%	Latent tuberculosis infection
				FVC: 92%	
				FEV_1_/FVC: 76%	
				DLCO: 96%	
CO-2	60–69	1980s (23 yrs)	Diffuse perilymphatic nodules; calcified mediastinal lymphadenopathy	FEV_1_: 57%^§^	Rheumatoid arthritis
				FVC: 48%^§^	
				FEV_1_/FVC: 91%	
				DLCO: 62%^§^	
CO-3	50–59	2000s (13 yrs)	Upper lung predominant nodules; calcified mediastinal lymphadenopathy	FEV_1_: 82%	Latent tuberculosis infection
				FVC: 82%	
				FEV_1_/FVC: 80%	
				DLCO: 102%	
CO-4	40–49	2000s (17 yrs)	Diffuse centrilobular nodules; upper lung ground glass opacities; calcified mediastinal lymphadenopathy	FEV_1_: 96%	None
				FVC: 92%	
				FEV_1_/FVC: 82%	
				DLCO: 74%^§^	
CO-5	50–59	1980s (23 yrs)	Upper lung predominant nodules; calcified mediastinal lymphadenopathy	FEV_1_: 105%	Rheumatoid arthritis
				FVC: 104%	
				FEV_1_/FVC: 80%	
				DLCO: 90%	
CO-6	40–49	1990s (22 yrs)	Upper and middle lung predominant nodules	FEV_1_: 105%	None
				FVC: 103%	
				FEV_1_/FVC: 82%	
				DLCO: 102%	
CO-7	40–49	1990s (24 yrs)	Upper lung predominant nodules; mild paraseptal emphysema; calcified mediastinal lymphadenopathy	FEV_1_: 90%	Rheumatoid arthritis
				FVC: 83%	
				FEV_1_/FVC: 86%	
				DLCO: 77%^§^	
TX-1	50–59	2010s (2 yrs)	Bilateral lower lobe ground glass opacities and scattered nodules	FEV_1_: 65%^§^	None
				FVC: 70%^§^	
				FEV_1_/FVC: 73%	
TX-2	50–59	1980s (31 yrs)	Multiple bilateral pulmonary nodules; ground glass opacities in lower lobes and calcified hilar lymphadenopathy	FEV_1_: 118%	None
				FVC: 115%	
				FEV_1_/FVC: 80%	
TX-3	50–59	1980s (31 yrs)	Upper lobe predominant reticular and partially calcified nodular opacities with bilateral partially calcified hilar and mediastinal lymphadenopathy	FEV_1_: 89%	None
				FVC: 102%	
				FEV_1_/FVC: 69%^§^	
TX-4	40–49	2010s (2 yrs)	Upper lobe predominant nodules with bilateral hilar and mediastinal lymphadenopathy	FEV_1_: 54%^§^	None
				FVC: 55%^§^	
				FEV_1_/FVC: 79%	
WA-1	30–39	2010s (6 yrs)	Diffuse, upper lung predominant nodules with early conglomeration; mediastinal lymphadenopathy	FEV_1_: 41%^§^	None
				FVC: 44%^§^	
				FEV_1_/FVC: 77%	
				DLCO: 32%^§^	

**Abbreviations**: CA = California; CO = Colorado; CT = computed tomography; DLCO = diffusing capacity for carbon monoxide; FEV_1_ = forced expiratory volume in 1 second; FVC = forced vital capacity; TX = Texas; WA = Washington.

* Exact years of employment suppressed for patient confidentiality.

^†^ Patient died from silicosis.

^§^ Abnormal pulmonary function test defined as FEV_1_<80% predicted, FVC<80% predicted, FEV_1_/FVC<70%, and DLCO <80% predicted. Global Lung Function Initiative reference values (2012) were used to calculate percentage predicted values for spirometry; DLCO was based on reference values in Crapo RO, Morris AH. Standardized single-breath normal values for carbon monoxide diffusing capacity. Am Rev Respir Dis 1981;123:185–9. For some cases, only spirometry was performed; therefore, DLCO is not reported.

^¶^ Silicosis diagnosed based on postmortem review of lung tissue.

Further investigation of patient CA-1’s place of employment, in collaboration with the California Division of Occupational Safety and Health, identified two additional silicosis cases among stone fabricators. The first patient (CA-2) was a Hispanic man who worked at the same company during 2003–2016 and died in 2018 at age 36 years. He had a history of rheumatoid arthritis with positive rheumatoid factor and cyclic citrullinated peptide antibodies. He was hospitalized in 2016 with respiratory symptoms and chest CT findings of silicosis but was lost to medical follow-up. After his death, investigators obtained lung tissue from autopsy, which showed silicotic nodules and alveolar proteinosis (indicating accelerated silicosis). The third case occurred in a Hispanic man aged 36 years who had worked at the company for 11 years and received a silicosis diagnosis in 2018 (CA-3). Since initiation of this investigation, three additional employees of the same stone fabrication company, all Hispanic men aged 35–59 years (CA-4, CA-5, and CA-6), have screened positive for silicosis by chest radiograph, with diagnoses subsequently confirmed by chest CT.

**Colorado**. In January 2019, a Colorado physician specializing in occupational lung disease observed an increasing number of silicosis cases in her practice and undertook a systematic review of electronic medical records for patients she had seen during June 2017–December 2018 with a silicosis diagnosis (ICD-10 code J62.8). Typically, the physician saw two cases of silicosis in a year; however, during June 2017–December 2018, seven cases of silicosis were identified (CO-1–CO-7), all among employees of stone fabrication companies (Table). Two workers were female, and all seven of the workers were Hispanic. They had worked at 12 Colorado companies during 1984–2018, most of which employed <50 workers. Five patients reported cutting, grinding, and polishing mainly engineered stone; two reported only bystander exposure to engineered stone dust during workplace housekeeping duties.

All seven patients had chest CT findings consistent with silicosis. Four had undergone diagnostic lung biopsy before occupational medicine referral. One biopsy was prompted by findings on chest CT, and three patients had received a rheumatoid arthritis diagnosis based on positive autoimmune serology testing and erosive joint disease with lung biopsies showing findings of silicosis. Two patients had latent tuberculosis infection diagnosed by positive interferon-gamma release assays and negative sputum cultures. Pulmonary function was abnormal in five patients; one had severe restrictive lung disease, and four had exertional hypoxemia indicated by arterial blood gas testing. Six patients had two or more chest images for comparison; five showed progressive silicosis evidenced by increased profusion of lung nodules over time. Patients were medically removed from any ongoing silica exposure and counseled on workers’ compensation and the need for long-term medical follow-up. The federal Occupational Safety and Health Administration and the Colorado Department of Public Health and Environment were informed of these cases as occupational sentinel health events needing follow-up to protect other potentially exposed workers.

**Texas.** During March–April 2019, the Texas Department of State Health Services received reports of an apparent cluster of silicosis cases among workers at an engineered stone countertop manufacturing and fabrication facility. Twelve cases were identified as meeting the National Institute for Occupational Safety and Health surveillance case definition for silicosis.^§^ Four of the 12 workers (TX-1–TX-4) had silicosis diagnoses confirmed by chest CT (Table); the remaining eight workers screened positive by chest radiograph but did not have confirmatory findings on chest CT. All four of the persons with confirmed silicosis were men aged 40–59 years; two were Hispanic, and two were non-Hispanic black. Three worked as fabricators, and one worked in engineered stone slab casting and stripping. Work tasks included cutting, sanding, gluing, and finishing engineered stone countertops. Pulmonary function testing was abnormal in two patients, with findings of moderate to severe restriction. 

**Washington**. In May 2018, Washington’s Occupational Respiratory Disease Surveillance Program, through routine surveillance of workers’ compensation data, identified a case of biopsy-confirmed silicosis in a Hispanic man aged 38 years who had worked in stone countertop fabrication during 2012–2018 (WA-1) (Table). His work tasks included cutting, polishing, and lamination of both natural and engineered stone. Chest CT demonstrated findings of silicosis, and lung biopsy found conglomerate areas of fibrosis and polarizable particles. Pulmonary function testing showed a severe restrictive defect and reduced diffusion capacity. He received a diagnosis of progressive massive fibrosis (the most advanced form of silicosis) and has had progressive lung function decline, necessitating referral for lung transplantation evaluation. Washington’s Division of Occupational Safety and Health was informed of this case and completed a workplace inspection.

## Discussion

Although silicosis outbreaks have been reported among engineered stone fabrication workers in other countries (*2*–*5*), only one such case has been reported previously in the United States (*7*). This report describes 18 additional cases of silicosis, including two fatalities, occurring in four states among mainly Hispanic stone fabrication workers who worked principally with engineered stone materials. As reported in other countries, most of the workers in this series (11 of 18) were aged <50 years, with severe, progressive disease. Engineered stone contains substantially more silica than does natural stone (>90%, compared with <45% in granite) (*6*), exposing workers to higher amounts of silica dust. In recent years, engineered stone countertops have become increasingly popular; quartz surface imports to the United States increased approximately 800% during 2010–2018.^¶^

In addition to silicosis, two patients had latent tuberculosis infection, and five had concurrent autoimmune disease; autoimmune disease has also been documented among workers in this industry in other countries (*8*). Silicosis was not suspected in several patients with autoimmune disease until they underwent lung biopsy, underscoring the importance of taking an occupational history in patients with autoimmune diseases to improve recognition of workplace silica exposure.

Silicosis is preventable through effective workplace exposure controls; in the stone fabrication industry, this can include tools equipped with water feeds and well-designed local exhaust ventilation, and, when needed, appropriate respiratory protection.** Updated occupational silica standards, with more stringent requirements for exposure prevention and monitoring, medical surveillance, and a lower respirable crystalline silica PEL of 0.05 mg/m^3^, have been implemented since 2016 at the federal and state levels.^††^

Despite availability of exposure controls and recent passage of more stringent silica standards, exposure control and medical surveillance for silicosis in the stone fabrication industry remain challenging. As of 2018, there were an estimated 8,694 establishments and 96,366 employees in the stone fabrication industry in the United States.^§§^ Many stone fabrication shops are small-scale operations that might face safety challenges, including limited awareness, expertise, and investment in exposure-control technologies, that can result in inadequate worker protection. In addition, many employees in this industry are Hispanic immigrants, who might be especially vulnerable to workplace health hazards because they might have fewer employment options and diminished access to medical care and face threat of retaliation if they report workplace hazards or file workers’ compensation claims (*9*). As a result, these workers might not seek medical attention until symptoms are severe and disease is advanced.

The findings in this report are subject to at least two limitations. First, requirements for employee medical screening under the silica standard have only recently been established in most jurisdictions; many at-risk employees likely have not been screened for silicosis. Second, public health surveillance for silicosis varies across jurisdictions; the cases described in this report were identified through record review from an individual clinical practice (Colorado), state-based respiratory disease surveillance using workers’ compensation (Washington) or hospital discharge data (California), and employer or health care provider reports to a public health agency (Texas). Without systematic screening and surveillance of all at-risk workers, prevalence of silicosis and its associated conditions in stone fabrication workers in the United States remains unknown.

Given mounting evidence of silicosis risk among stone fabrication workers, the government of Queensland, Australia, initiated screening in 2018 for all at-risk employees. Ninety-eight cases of silicosis have been identified among 799 workers (12%) examined (*10*). These findings suggest that there might be many more U.S. cases that have yet to be identified.

Silicosis is preventable; the cases reported here highlight the urgent need to identify stone fabrication workers at risk and prevent further excess exposure to silica dust. Stone fabrication employers should be aware of this serious risk to their employees’ health and ensure that they adequately monitor and control exposures in compliance with the updated silica standards. To identify silicosis among already-exposed workers, employers should conduct required medical surveillance, and both employers and health care providers should notify appropriate public health agencies when cases are identified. State health departments and CDC can work together to standardize and improve public health surveillance for silicosis across jurisdictions. Effective disease surveillance and regulatory enforcement are crucial to address the emerging silicosis threat in the stone fabrication industry.

**Summary**

**What is already known about this topic?**

Respirable crystalline silica exposure causes silicosis, a disabling and sometimes fatal lung disease. Clusters of cases have been reported internationally among stone countertop fabrication workers, but only one U.S. case in this industry has been reported previously.

**What is added by this report?**

Eighteen cases of silicosis, including two fatalities, are reported among stone fabrication workers in four states. Several patients also had autoimmune disease and latent tuberculosis infection.

**What are the implications for public health practice?**

Stone fabrication workers, especially those working with engineered stone, are at risk for silicosis. Given the serious health hazard and significant number of workers at risk, additional efforts are needed to reduce exposures and improve disease surveillance.

## Footnotes

† A permissible exposure limit (PEL) is the highest permissible level of exposure for a specific substance for an employee, as established under state or federal occupational safety and health regulations. The PEL cited here is for exposure as an 8-hour time-weighted average, which represents an employee’s average airborne exposure to a particular substance during an 8-hour work shift.

§
https://www.cdc.gov/niosh/topics/surveillance .

¶
https://dataweb.usitc.gov/ .

** Additional information regarding controlling silica dust exposures is available at https://www.cdph.ca.gov/silica-stonefabricators and at https://www.cdc.gov/niosh/topics/silica/.

§§ Data from the Bureau of Labor Statistics quarterly census of employment and wages (https://www.bls.gov/cew/data.htm) for North American Industrial Classification System (NAICS) industry code 327911 (Cut Stone and Stone Product Manufacturing) and NAICS code 423320 (Masonry Material Merchant Wholesalers). At time of access, data for 2018 were preliminary.
